# Physicochemical characterization and microbiology quality of the *Pentadesma butyracea* fruit pulp collected from various parks in Benin

**DOI:** 10.1038/s41598-021-96211-z

**Published:** 2021-08-23

**Authors:** Bernolde Paul Ayegnon, Ifagbémi Bienvenue Chabi, Folachodé Ulrich Gildas Akogou, Adéchola Pierre Polycarpe Kayodé

**Affiliations:** 1 Laboratoire de Valorisation et de Gestion de la Qualité des Bio ingrédients Alimentaires (LABIO), Faculté des Sciences Agronomiques, Université d’Abomey-Calavi, Jericho, 03 BP 2819 Cotonou, Benin; 2 Département de Nutrition et Sciences Agro-Alimentaires (NSAA), Faculté d’Agronomie, Université de Parakou, Parakou, Benin

**Keywords:** Biochemistry, Microbiology

## Abstract

In Benin, *P. butyracea* fruit pulp undergoes significant post-harvest loss due to its low valorization. The collected fruits in five parklands from a survey of transformer's perception were characterized through their visual observations, the determination of their dimensions and pulp proportions. The nutritional value of pulp was evaluated through its chemical characterization. The results of this study revealed that four shapes (ellipsoid, globular, ovoid, ellipsoid twisted) identify the physical aspect of *P. butyracea* fruits in the *P. butyracea* parklands. The average length, diameter at the equator, mass and pulp proportion of fruits were 130.93 mm, 86.98 mm, 125.63 g and 35.79%, respectively. The physicochemical characterization showed that the *P. butyracea* fruit pulp contained 3.37–3.41 pH, 3.33–4.61% protein, 20.37–20.78% fiber, 2.78–3.57% ash, 6.31–6.51% crude lipid, 85.77–86.47% moisture, 21.54–23.22 mg/mL total phenolics, 0.73–1.03% titratable acidity, 65.10–66.94% carbohydrates, 335.75–338.99 kcal calorie and minerals (Ca, Fe, Zn, Mn). The antioxidant activity result showed that the *P. butyracea* fruit pulp is a valuable source of antioxidant pigments. Faecal coliforms are not detected in pulps and the aerobic mesophilic bacteria, yeasts and moulds, and *Staphylococcus aureus* were below detection level in *P. butyracea* fruit pulps.

## Introduction

*Pentadesma butyracea* Sabine (Clusiaceae) is a ligneous forest species with multipurpose uses. It is widely distributed in Africa from Guinea-Bissau to thewest of the Democratic Republic of Congo^1^. *P. butyracea* is found in the centre and northern part of Benin in forest galleries and along water ways^2,3^. The *P. butyracea* tree gives a fruit, made up of pulp and seeds. The seeds are the element of the fruit more nowadays exploited because it contains an almond rich in butter. The rural population used *P. butyracea* butter in cosmetic and in the manufacturing of soap^4^. This butter is mostly used for food preparation, cosmetic and therapeutic applications because of its yellow colour, hard texture, relatively sweet taste, softening, lubricating and healing qualities^5^. Comparative studies carried out by Ref.^5^ on *P. butyracea* and shea butters, showed that *P. butyracea* butter is clearly better than shea butter mostly on the organoleptic level. Reference^6^ evaluated the physicochemical characteristics of *P. butyracea* butter and reported that the oil content was 419.0 g/kg, crude protein content was 44.0 g/kg, lysine content was 3.2 g/kg dry matter, methionine content was 1.6 g/kg and cysteine was 1.5 g/kg.

In spite of the strong knowledge of the biochemical composition of the *P. butyracea* kernels and potential application of its butter in food, in cosmeic and therapeutic field, there is no information on the physicochemical and biactive properties, microbiological and sensorial characteristics of *P. butyracea* fruit pulp. During *P. butyracea* butter extraction, the fruit pulp undergoes significant loss because of the ignorance of the nutritional value and bioactive properties of this part of the fruit. This pulp is almost unutilised, unexploited for several reasons. One of the reasons is the ignorance by the populations of the nutritional value of pulp. More than 99% of this pulp are thrown by the gatherers in spite of its potentialities. Studies carried out on the pulp of the shea fruit from Chad^7^, Nigeria^8^ and Uganda^9^, showed that it contains a significant quantity of nutritive elements such as the glucides, the proteins, the fibres, minerals and the vitamins. It is then necessary to develop technologies of valorization of *P. butyracea* fruit pulp in Benin. For this reason, it is important to study the nutritional quality of the fruits pulp in Benin. The present study evaluated the nutritional value and microbiology quality of the *P. butyracea* fruit pulp collected from various parks in Benin and determined the various uses of fruit pulp using a quantitative survey approach and the Physical characterization of the fruit.

## Materials and methods

### Survey and data collection

The study was undertaken in five municipalities of nord Benin, namely Bassila, Tchaourou, Toucountouna, Natitingou and Kandi. An investigation was conducted between May and June 2020 in villages having a tradition of *P. butyracea* fruits processing. All participans were more than 18 years, selected from the study sites. The study interest and procedure were explained to the community by village leaders. Therefore, all participans approved the activity because they understood that it will allow them to valorize the *P. butyracea* fruit pulp. Each participant provided informed consent prior to responding to the survey. Individuals who did not provide informed consent could not proceed with the survey. Also, informed consent was obtained from a legal guardian on behalf of illiterate participants. This survey was approved by the Institution Ethics Board and the School of Nutrition and Food Sciences and Technologies (SNFST) of Abomey-Calavi University (UAC), approval N° F-025-2020-SNFST. To determine adequate number of respondents for our survey among the *P. butyracea* actors per parkland, the following formula was used: Ni = 4Pi(1 − Pi)/d2 where Ni is the total number of *P. butyracea* actors to be surveyed in parkland i, Pi is the proportion of *P. butyracea* actors found in previous studies in parkland i, and d is the expected error margin in the conclusion, which was fixed at 0.1^10^. Based on this method, a total of 260 subjects distributed in twelve villages of aforementionned municipalities was obtained. Data were collected by administering questionnaires to randomly selected and willing respondents. The questionnaires included the following aspects: *P. butyracea* actor’s perception on fruit pulp, various uses, collection state and various methods of collection of the fruits of *P. butyrace.* In addition, the various fruit shapes available in each commune was determined. This research was performed in accordance with relevant guideline for humans and regulations.

### Subjects

The socio-demographic characteristics of the participants in the quantitative survey were as follows. Subjects were 180 females and 80 males. They were processors of *P. butyracea* butter. Their socio-cultural origin was diverse. Most of them were Nago (20%), Ditamari (19.23%), Anii (15.38%), Kotocoli (11.54%), Waama (14.23%), Boo (13.46%) and Fulani (6.15%). Sixty-five percent of the respondents were 35–50 years old, 29.45% between 20 and 34 and 5.87% over 51. Their educational background was poor, as 79.5% of them were illiterate and only 20.5% had attended primary school. This reflects the general education level in the country. They practise different religions, i.e. practitioners of endogenous religions (7.7%), Muslims (30.2%) and Christians (62.1%).

### Samples collection

The fruits of *P. butyracea* were collected from May to June 2020 in the forests galleries of four communities in northern Benin that is Tchaourou (8° 45′–9° 20′ N and 2° 10′–3° 40′ E), Kandi (11° 7′–11° 43′ N and 2° 56′–2° 13′ E), Toucountouna (10° 20′–10° 45′ N and 1° 10′–1° 40′ E) and Bassila (8° 30′–9° 30′ N and 1° 00′–2° 30′ E). In each parkland the fruits were collected under ten *P. butyracea* tree packed in jute bags and transported to the laboratory. The fruits from each site were then mixed together. The fruits were depulped and fruit pulp were mixed for different chemicals analyses. Experimental research and feld studies on plants (either cultivated or wild), including the collection of plant material have complied with relevant institutional, national, and international guidelines and legislation. All methods were performed in accordance with the relevant guidelines and regulations.

### Physical charecterization of the *P. butyracea* fruits

The fruits evaluation has been done immediately after the sample collection. The characteristics analysed were: diameter, length, fruit mass and fruit pulp mass. Diameter and length (mm) of the fruits were measured with a precision caliper (0.01 mm) and mass of the fruits (fm) and pulps mass (pm) were determined using balance with precison is 1/10.000. Measurements were performed on 50 fruits chosen at random per sample and by shape (ellipsoid, globular, ovoid and ellipsoid twisted) from each park. The generated values allow calculating the proportion of pulp (P) with P(%) = pm/fm × 100.

### Evaluation of physicochemical composition of *P. butyracea* fruit pulp

The proximate analysis of fruit pulp samples were analyzed using standard methods^11^. The moisture content was determined by drying about five grams of the fresh sample in an oven at 105 °C to constant weight. Crude ash was determined by incineration of 5 g of sample in a muffle furnace at 550 °C for 8 h. The nitrogen (N) content was estimated by micro-Kjeldahl method and crude protein content calculated as N% × 6.25. Crude lipid was exhaustively Soxhlet extracted from 5 g sample with nhexane for 4 h. Crude fibre content was determined by treating 2 g sample with 1.25% (W/V) H_2_SO_4_ and 1.25% (W/V) NaOH. Minerals (calcium, magnesium, iron and zinc) of the samples were then analyzed with Atomic Absorption Spectrophotometer model Analysit-400. The pH was determined using a digital pHmeter (HI8418; Hanna instruments, Limena, Italy) calibrated with buffers at pH 4.0 and 7.0 (WTW, Weilheim, Germany). The titratable acidity, expressed as lactic acid, was carried out using the method described by Ref.^12^. The Brix values were carried out using a refractometer. The available carbohydrate (CHO) was calculated by difference. Calorific value (CV) was determined using the following equation:

$$ {\text{CV }}\left( {{\text{kcal}}/{1}00{\text{ g}}} \right) \, = \, \left( {{\text{CHO }} \times { 4}} \right) \, + \, \left( {{\text{CL }} \times { 9}} \right) \, + \, \left( {{\text{CP }} \times { 4}} \right){\text{ with CL}} = {\text{ Lipides}},{\text{ CP}} = {\text{ Proteines}} $$

### Microbiological characteristics of *P. butyracea* fruit pulp

Measurements were performed on 10 fruits chosen at random per sample. Fruit pulps samples were analyzed for total bacterial count, total coliforms, thermotolerant coliforms, yeasts and moulds and *Staphylococcus aureus* according to standard methods of Ref.^13^. Briefly, 10 g of fruit pulps were weighed into sterile bags and homogenized in 90 mL of sterile peptone water (0.1%) and NaCl (0.85%). Serial dilutions were made in BPW. Aerobic mesophilic bacteria were analysed using plate count agar after incubation at 30 °C for 72 h, total coliforms were enumerated on violet red bile agar after incubation at 30 °C for 24 h), thermotolerant coliforms on Violet Red Bile Agar at 44 °C for 24 h, yeasts and moulds on malt extract agar at 25 °C for 72 h. *S. aureus* determined on Baired Parker agar supplemented with egg yolk and the plates incubated at 37 °C for 48 h.

### Preparation of extracts for the determination of total phenolics and antioxidant activity

Samples were extracted in methanol/HCl (99:1 (v/v) following the method described by Ref.^14^. Fifty milligrammes (50 mg) of each sample were extracted at room temperature with 1.5 mL of solvent under agitation using a magnetic stirrer for 30 min. The mixtures were centrifuged at 2500*g* for 10 min and the supernatants were collected. The residues were re-extracted twice under the same conditions, resulting in 3 mL crude extract. All extracts were used as they were after centrifugation for various analyses.

### Total phenolics determination

Total phenolics were measured following the method of Ref.^15^ modified by Ref.^14^ as follows: to 300 μL of extract, 4.2 mL of distilled water, 0.75 mL of Folin-Ciocalteu’s reagent (Merck, Germany) and 0.75 mL of sodium carbonate solution (200 g/L) were added. After incubation for 30 min, the optical density was measured at 760 nm against a blank. Gallic acid was used as standard and the results were expressed as gallic acid equivalent (GAE) per g of sample DM.

### Antioxidant activity determination

The antioxidant activity was measured using DPPH (2,2-diphenyl-1-picrylhydrazyl). The DPPH method was conducted by adaptation as described by Scherer and Godoy^16^. Equal volumes (100 μL) of DPPH (50 μM) and plant extracts (12.5 mg/mL) were mixed and incubated for 20–30 min in the dark at room temperature. Then, the absorbance was read at 517 nm and the blank was a mixture of methanol and DPPH (v:v). This activity was given as % DPPH scavenging and calculated using Eq. (1):

1 $$ \% {\text{Inhibition DPPH}}^{.} = \frac{{A_{control} - A_{sample} }}{{A_{control} }} \times 100, $$

where A_control_ is absorption of DPPH solution, and A_sample_ is absorbance of the test sample. The half maximal effective concentration (EC_50_) is the amount of sample necessary to decrease the absorbance of DPPH by 50%. It was determined graphically using a calibration curve in the linear range by plotting the extract concentration and the corresponding scavenging effect. Ascorbic and gallic acids were used as positive controls. All the analyses were carried out in triplicate.

### Statistical analysis

All data were expressed as mean ± standard deviation (n = 3 replicates). Data were analyzed using one-way ANOVA using SPSS 16.0. Duncan’s multiple-range test was used to determine the difference between means. A significant difference was considered at the level of p ≤ 0.05.

### Collection of *P. butyracea* fruits

Fruits were collected in five muicipalities of nord Benin under permission from Department of the Environment of National Forestry Commission, approval N°2020-FOR-03-093-PENESSOULOU. Sometimes, there are the foresters who lead us in the parks to collect the *P. butyracea* fruits.

### Experimental research and field studies on plants

All experimental protocol and methods was approved by the Faculty of Agronomics Sciences (FSA) of Abomey-Calavi University (UAC), approval N°202040016. The fruits have been collected between May and June 2020 in five muicipalities of nord Benin, and transported to food laboratory of School of Nutrition and Food Sciences and Technologies of FSA for analyze. Moreover, an investigation was conducted between May and June 2020 in the villages retained as focus group. It was carried out through a guide of interview pre-established. This survey was approved by the School of Nutrition and Food Sciences and Technologies (SNFST) of FSA, approval N° F-025-2020-SNFST. Data were collected by administering questionnaires to randomly selected and willing respondents. To determine adequate number of respodents for survey, method according^10^ used.

## Results and discussion

### Intercultural convergence uses of *P. butyracea* fruit pulp in Benin

The different uses of *P. butyracea* fruit pulp by rural households in the study zones are presented in Table 1. Among the different ethnic groups involved in the *P. butyracea* fruit pulp use, the Fulani reported to know about trees producing fruits with bitter pulp and their yellow color. The fruit pulp sometimes is consumed by the farmers in the fields. *P. butyracea* fruit pulp is used mainly in the therapeutic applications and less in cosmetic and food sector. All the socio-cultural groups interviewed use the pulp for therapeutic applications. More specifically, *P. butyracea* fruit pulp is used by Anii (100% of respondents), Kotocoli (96.80% of respondents), Boo (97.30% of respondents) and Waama socio-cultural groups (94.30% of respondents) in the treatment of digestive system disorders such as bloated stomach and constipation. Reference^2^ reported that the fruits are used to treat the digestives disorders, the genito-urinary infections and also in the human consumption. Apart from this traditional utilization, *P. butyracea* pulp is also used in locally produced soap and cosmetics. For instance, the Kotocoli (89.50% of respondents), Waama (77.6% of respondents) and Otamari groups (67.8% of respondents) used it in chiropodist and local soap preparation. Moreover, all socio-cultural groups interviewed use pulp to wash their pans, they also use it against rust. According the *P. butyracea* butter producers, the fruit is available from May to June. The period of abundance is the end of May in most of the parks. It are available at the beginning of the rainy season, a period characterized by general food scarcity in Sub-Saharan regions. Gathering of *P. butyracea* fruits is generally done by women and children early in the morning. They collect under the trees all fruits found without sorting. After gathering, the fruits are depulped in the field and kernels brought back home.

**Table 1:** Food, cosmetic and therapeutic uses of *P. butyracea* fruit pulp by different socio-cultural groups (percent of producers surveyed, N = 260).

Socio-cultural group	Type of use		
	Food	Therapeutic	Cosmetic
Boo (n = 35)	8.45	97.30	0
Nago (n = 52)	2.63	45.20	0
Anii (n = 40)	4.25	100	7.5
Otamari (n = 50)	3.2	92	67.8
Kotocoli (n = 30)	2.15	96.80	89.5
Waama (n = 37)	2.10	94.30	77.6
Fulani (n = 16)	1.7	88.5	35.7
Total (N = 260)	3.50	87.73	39.73

*n* number of respondents.

### Physical characteristics of *P. butyracea* fruits

**Figure 1:**
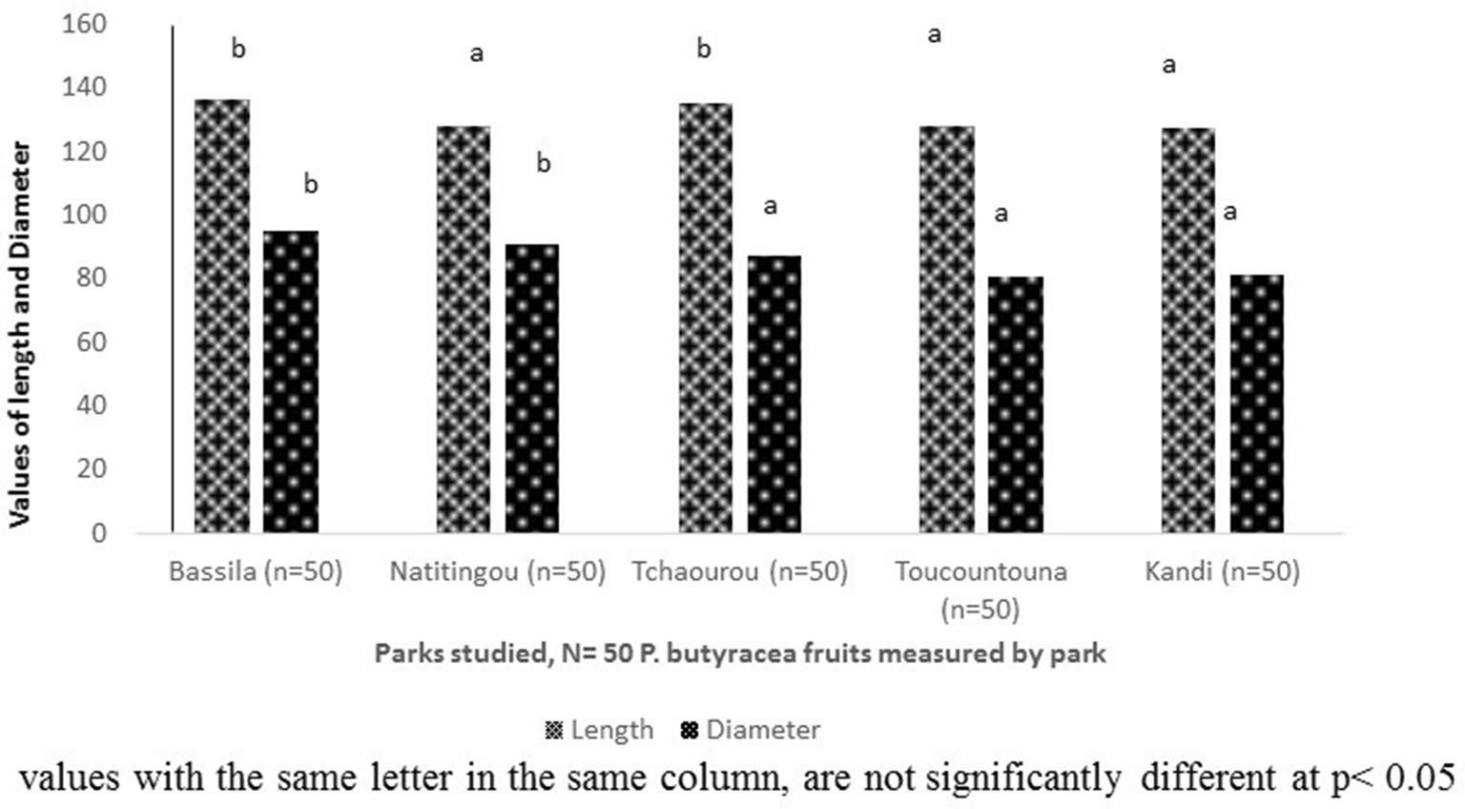
Dimensions of *P. butyracea* fruits by park collected in June 2020 in Benin.

**Figure 2:**
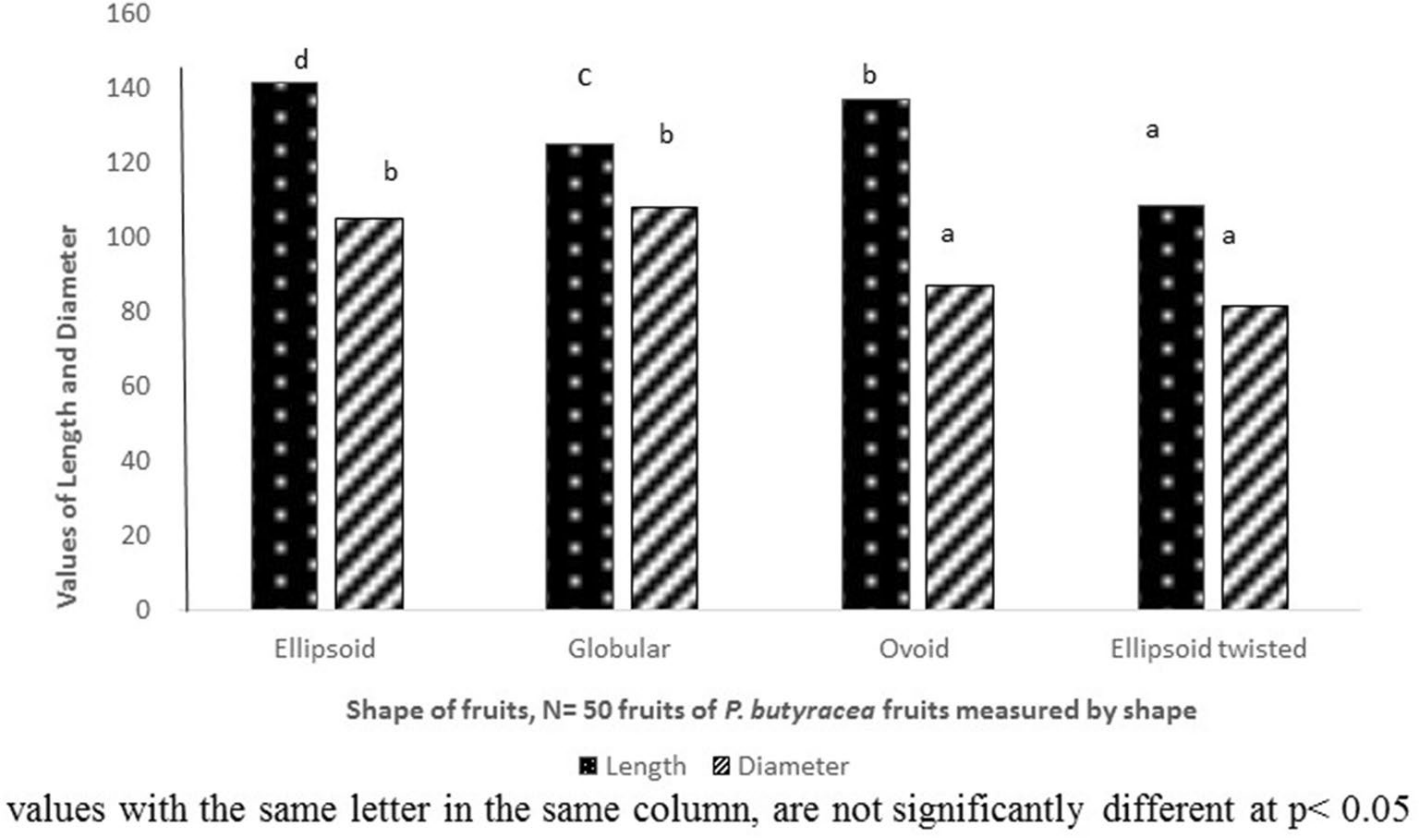
Dimensions of *P. butyracea* fruits by shape collected in June 2020 in Benin.

The dimensions of *P. butyracea* fruits from various parks in northern of Benin were presented in the Fig. 1. The length of the fruits ranged from 127.31 to 136.16 mm, with an average value of 130.93 mm. Also, the diameter of the fruits ranged from 80.42 to 95.03 mm, with an average value of 86.98 mm. There was a significant difference between parks for these parameters (p ≤ 0.05). Thus, the lowest length and diameter of fruits were recorded on the fruits collected in Kandi, Toucountouna and Natitingou, while the fruits with the highest values were originated from Bassila and Tchaourou. Clearly, there is a big variation within the fruits of *P. butyracea* in terms of their morphological characteristics. The major factors that could affect these characteristics are the plant age, its genotype and its growing environment. The dimensions of different shapes of *P. butyracea* fruits from various parks in northern of Benin were presented in the Fig. 2. The dimensions (length and diameter) of *P. butyracea* fruits varies significantly (p ≤ 0.001) between shapes and the length of *P. butyracea* fruits shapes ranged from 108.39 to 141.48 mm, with a mean value of 128.01 mm. In addition, the diameter of fruits shapes ranged from 81.45 to 108.06 mm, with an average value of 95.41 mm. The highest length and diameter of fruits were recorded to the ellipsoid and globular shapes, while the lowest lengtth and diameter of fruits were found in the ellipsoid twisted shape. This result is in agreement with characteristics of fruits described by collectors during the survey. The collectors of *P. butyracea* fruits affirmed that the fruits having the ellipsoid and globular shapes are of big size. In addition, more than 70% of the fruits collected in the various parks are ovoid or globular forms.

The mass of fruit, mass and proportion of pulp from various parks are presented in Table 2. The mass of fruit from different parks ranged from 343.27 to 475.35 g, with a mean value of 403.30 g. In the same way, the mass and proportion of pulp ranged from 95.98 to 162.50 g, with an average value of 125.63 g, and 32.76 to 39.12%, with a mean value of 35.79%, respectively. There was a significant difference between the parks studied for fruits mass, pulp proportion and pulp mass (p ≤ 0.001). Thus, the highest fruit mass, pulp proportion and pulp mass were found on the fruits collected in Bassila and Tchaourou parks, while the lowest values of these parameters were observed on the fruits from Kandi and Toucountouna parks. The major factors that could affect these characteristics of fruits are rainfall. Thus, Tchaourou and Bassila are localised in the Sudano-Guinean transitional climatic zone with an average rainfall varying from 850 to 1850 mm and 1000 to 1300 mm, respectively. Kandi and Toucountouna are located in the Sudanian climatic zones with an average rainfall of 1200 and 1000 mm, respectively^5,17^. Moreover, fruit mass, pulp proportion and pulp mass varies significantly (p ≤ 0.001) between fruits shapes (Table 3) and ranged from 241.50 to 535.25 g, 81.75 to 204.50 g and 30.44 to 36.74%, respectively. The highest fruit mass, pulp proportion and pulp mass of fruits were recorded to the ellipsoid, ovoid and globular shapes respectively, while the lowest values were found in the ellipsoid twisted shape. The pulp proportion obtained was lower compared to the range of 57–70% for shea fruit pulp collected from various parks in Benin^18^. The ovoid shape has the proportion of pulp more significant (36.74%) which is definitely higher than those of the ellipsoid twisted (33.30%), ellipsoid (33.05%) and globular shape (30.44%).

**Table 2:** Proportion of *P. butyracea* fruit pulp by park collected in June 2020 in Benin.

Parks	Fruit mass (g)	Pulp mass (g)	Proportion of pulp (%)
Bassila	475.35 ± 91.13C	162.50 ± 61.13d	39.12C
(n = 50)	[242–705]	[94.34–304]	[23.54–42.53]
Natitingou	389.11 ± 71b	128.75 ± 77.13b	35.71b
(n = 50)	[144–654]	[30–354]	[12.29–43.16]
Tchaourou	461.25 ± 74.04C	143.15 ± 76.62C	37.71b
(n = 50)	[259–712]	[67–194]	[29.23–46.06]
Toucountouna	347.51 ± 56.31a	97.75 ± 58.82a	33.67a
(n = 50)	[123–497]	[45–121]	[25.89–40.14]
Kandi	343.27 ± 35.17a	95.98 ± 47.41a	32.76a
(n = 50)	[113–484]	[38–129]	[28.37–41.21]
Average	403.30	125.63	35.79
CV^2^	15.42	22.97	7.45

Mean ± Standard deviation; n = number of fruits included; values with the same letter in the same column, are not significantly different at p < 0.05.

*CV*^2^ Coefficient of variation. In bracket [minimum value–maximum value] of the 50 fruits.

**Table 3:** Proportion of *P. butyracea* fruit pulp by shape collected in June 2020 in Benin.

Shapes	Fruit mass (g)	Pulp masse (g)	Proportion of pulp (%)
Ellipsoid	535.25 ± 97.34d	204.50 ± 61.77d	33.05a
(n = 50)	[342–703]	[100–304]	[21.54–40.54]
Globular	459.5 ± 94c	158.75 ± 87.19c	30.44a
(n = 50)	[244–702]	[30–303]	[12.29–43.16]
Ovoid	411.25 ± 74.04b	137.25 ± 96.66b	36.74b
(n = 50)	[259–541]	[67–199]	[29.23–46.06]
Ellipsoid twisted	241.50 ± 60.36a	81.75 ± 31.82a	33.30a
(n = 50)	[161–297]	[45–121]	[25.99–40.74]
Average	411.87	145.56	33.38
CV^2^	30.23	35.00	7.74

Mean ± Standard deviation; n = number of fruits included; values with the same letter in the same column, are not significantly different at p < 0.05.

*CV*^2^ Coefficient of variation. In bracket [minimum value–maximum value] of the 50 fruits.

### Nutritional compositions of *P. butyracea* fruit pulp

The data on the chemical composition of the *P. butyracea* fruit pulp, as determined, are presented in Table 4. The moisture content of the *P. butyracea* fruit pulp ranged from 85.77 to 86.47%, with no significant different between the *P. butyracea* parks (p > 0.05). These values were similar to those of the pineapple (87%) and apple (86%)^19^ and shea fruit pulp from various zones in Benin (77.56–80.89%) reported by Ref.^18^. The *P. butyracea* fruit pulp would not be microbiologically stable at this moisture content as it would allow the proliferation of microorganisms. The ash content of the pulps was comparable to that of the bush mango fruit pulp (02.10%)^20^ but higher than the 0.33–0.71% reported for shea fruit pulp jam^21^.This is indicative of the high mineral content of *P. butyracea* fruit pulp. The pH of the pulps ranged from 3.27 to 3.41. The pH of the different pulps is not significantly different (p > 0.05). The low pH of the *P. butyracea* fruit pulp would not create favorable conditions for many organisms to sporulate and multiply^20^. The low pH and high acidity for these products was due to the organic acids in the fruit pulp^22^. These properties would confer long shelf life on the products by hindering proliferation of undesirable microorganisms^23^. The titratable acidity of the *P. butyracea* fruit pulp from different park in Benin ranged from 0.73 to 1.03%, with significant different between the parks. The highest value of titratable acidity was found in the samples from Kandi park while the lowest values of this parameter was observed in the samples from Bassila park. The value for the titratable acidity was similar to that reported by Ref.^19^ in orange/pineapple juice blends (0.47–1.27%), but higher than 0.233–0.330% obtained by Ref.^21^ in shea fruit pulp juice. The acids present in food not only improve its palatability but also influence its nutritive value^20^. The proteins value of fruit pulp from different parks ranged from 3.33 to 4.61% dw. There was a significant difference (p ≤ 0.05) between the parks studied for this parameter. These proteins values were lower than that of the shea fruit pulp (3.96–6.34%) reported by Ref.^18^, but is higher than 1.5–3.5% obtained by Ref.^9^ in Uganda and 1.09% reported by Ref.^24^ from the fresh shea pulp. In addition, Protein content of *P. butyracea* fruit pulp was similar to that found in mango pulp (4.8%) reported by Ref.^20^. The differences in the protein contents may be due to environmental differences in areas of production. The crude fiber contents of the pulp ranged from 20.37 to 20.78%. The values obtained for the crude fiber content of the pulp from the five different localities was very close, with no statistically significant difference (p > 0.05). The value of crude fiber content for *P. butyracea* fruit pulp of 20.37–20.78% is within the crude fiber values of most wild and domesticated fruits and higher than in legumes with mean values ranging between 5 and 6%^25^. In addition, these values are high compared to the fiber value for bush mango pulp of 2.5% reported by Ref.^20^, and high than the shea fruit pulp collected from various parks in Benin with values ranging between 5.97 and 6.52% as reported by Ref.^18^. The highest crude fiber content of the *P. butyracea* fruit pulp makes it digestible beverage for children as reported by Ref.^26^ for juices made from citrus fruits. It also implies that promoting consumption of *P. butyracea* fruit pulp is of great benefit to the human diet. The crude lipids content of *P. butyracea* fruit pulp ranged from 6.31 to 6.51%, with no significant different between the *P. butyracea* parks (p > 0.05). These values were higher than those of the shea fruit pulp from different shea zones in Uganda (1.5–3.5%) reported by Ref.^9^, and 1.4% obtained by Ref.^20^ in bush mango fruit pulp. The values for carbohydrates ranged from 65.10 to 66.94% and showed no significant difference (p > 0.05) between the samples. This is in close relationship with the range 80.8% reported by Ref.^20^ for bush mango fruit pulp. The carbohydrate value was higher when compared with values 7.3–9.9 g/100 g obtained by Ref.^27^ in *Butyrospermum paradoxum* fruit pulp and 12.6–19.4 g/100 g reported by Ref.^9^ for shea fruit pulp in different shea districts of Uganda. The *P. butyracea* fruit pulp was rich in carbohydrate when compared with other nutrients of the fresh pulp. The energy content of the pulp varies from 335.75 to 338.99 kcal, and shows no significant different (p > 0.05) between sites. The highest fat and carbohydrate contents of the *P. butyracea* pulp contributed to the higher energy content of the pulp. The energy obtained from *P. butyracea* pulp was higher than that of 1289.2 kj/100 g provided by the jams processed from shea fruit pulp reported by Ref.^21^.

**Table 4:** Chemical composition of *P. butyracea* fruit pulp by park collected in June 2020 in Benin.

Parameters	Bassila	Natitingou	Tchaourou	Toucountouna	Kandi
Moisture (%)	85.88 ± 1.23a	86.47 ± 2.05a	85.77 ± 3.12a	85.94 ± 2.13a	86.44 ± 1.27a
pH	3.37 ± 0.28a	3.27 ± 0.72a	3.34 ± 0.73a	3.41 ± 0.76a	3.39 ± 0.46a
Acidity titratable (%)	0.73 ± 0.34a	0.82 ± 0.12b	0.77 ± 0.43a	0.88 ± 0.27b	1.03 ± 0.55c
Crude fiber (%) dw	20.38 ± 0.57a	20.78 ± 0.53a	20.51 ± 0.34a	20.37 ± 0.71a	20.49 ± 0.63a
Crude Proteins (%) dw	3.33 ± 1.61a	4.61 ± 1.46b	3.65 ± 2.11a	4.45 ± 1.23b	3.36 ± 2.24a
Crude lipids (%) dw	6.38 ± 0.19a	6.31 ± 0.17a	6.43 ± 0.21a	6.51 ± 0.77a	6.46 ± 0.63a
Carbohydrate (%) dw	66.94 ± 0.16a	66.13 ± 0.78a	66.63 ± 0.88a	65.10 ± 0.93a	66.71 ± 0.67a
Calorie (kcal) dw	338.50 ± 0.63a	335.75 ± 0.47a	338.99 ± 0.56a	336.79 ± 0.74a	338.42 ± 0.71a
Ash (%) dw	2.97 ± 0.25a	3.17 ± 2.11b	2.78 ± 1.57a	3.57 ± 0.77b	2.98 ± 1.64a
Fe (µg/g)	176.44 ± 0.76c	198.03 ± 0.34a	194.57 ± 0.77b	205.53 ± 0.44d	193.37 ± 0.41b
Ca (µg/g)	1866.75 ± 0.23b	1871.73 ± 0.12b	1793.71 ± 0.53a	1921.75 ± 0.77c	1798.45 ± 0.71a
Zn (µg/g)	16.82 ± 0.33a	18.84 ± 0.23b	21.45 ± 0.12c	19.07 ± 0.67b	20.87 ± 0.57c
Mn (µg/g)	42.13 ± 0.54b	49.51 ± 0.83a	40.71 ± 0.71d	50.31 ± 0.47d	46.77 ± 0.36c

Mean ± Standard deviation; values with the same letter in the same line, are not significantly different at p < 0.05.

The minerals profile of the *P. butyracea* fruit pulp result was presented in Table 4. The most predominant mineral found in the *P. butyracea* fruit pulp was calcium with values ranged from 1793.71 to 1921.75 µg/g. The highest value of calcium was found in the samples from Toucountouna park while the lowest values of this mineral was observed in the samples from Tchaourou park. The calcium content was higher when compared with that of shea fruit pulp in Uganda (37.20–95.60 mg/100 mL) reported by Ref.^9^ and mango fruit pulp (190 mg/100 mL)^20^. In addition, iron and manganese were also high with values ranged from 176.44 to 205.53 µg/g and 40.71 to 50.31 µg/g respectively. Iron and manganese were highest in the samples from Toucountouna park and the lowest value in the samples from Bassila and Tchaourou parks, respectively. The iron content was higher than 2.5 mg/100 g obtained by Ref.^20^ in mango fruit pulp and 27.65 µg/kg reported by Ref.^24^ from fresh shea fruit pulp in Ghana. Manganese content found in *P. butyracea* fruit pulp was higher than 12.50 µg/kg obtained by Ref.^24^ from fresh shea fruit pulp in Ghana.

### Microbial characteristics of *P. butyracea* fruit pulp

Hygiene quality of *P. butyracea* fruit pulp samples from various parks in Benin was presented in Table 5. Samples of *P. butyracea* fruit pulp had various microbial load with low count of aerobic mesophilic germs (1.70–2.75 log10 CFU/g), yeasts and moulds (1.85–2.97 log10 CFU/g), total coliforms (1.11–2.37 log10 CFU/g) and *staphylococcus aureus* (1.05–1.37 log10 CFU/g) (Table 1). The number of aerobic mesophilic bacteria in all collected samples was close to the international standard of 4 log10 CFU/g^28,29^. However, the highest microbial count was found in sample collected in Tchaourou and Kandi parks, while the lowest microbial count was observed in fruit pulp from Bassila, Naitingou and Toucountouna. Difference between parks for the number of yeasts and moulds (p ≤ 0.001) and total coliforms (p ≤ 0.001) were observed, with samples collected from Tchaourou park giving significantly higher values of yeasts and moulds compared with the samples from other parks. Water and environment may play a major role in the yeasts and moulds contamination of *P. butyracea* fruit pulp especially during washing of fruits. The highest count of yeasts and moulds is also due to the acidic nature of *P. butyracea* fruit pulps which probably favours the growth of yeasts. Moreover, no faecal coliforms were detected in all fruit pulps. Furthermore, there is no significant difference in *Staphylococcus aureus* counts between the fruit pulp collected in various parks (p > 0.05). The presence of *Staphylococcus aureus* in the fruit pulp is attributed to its wide spread in the environment. The primary habitat of these organisms are the interior body and skin of man and animal from where this microbes are transferred to the fruits and subsequent transfer to the fruit pulp during treatment. However, the level of all microbial enumerated in *P. butyracea* fruit pulps was under the detection limit. The acidity of pulps could be a factor that limited the development of spoilage and pathogen microorganisms in pulps.

**Table 5:** Microbiological qualiies of *P. butyracea* fruit pulp by park collected in June 2020 in Benin.

Microorganisms (log_10_ UFC/g)	Microbiological qualiies of pulp				
	Bassila	Tchaourou	Kandi	Natitingou	Toucountouna
Aerobic mesophilic bacteria	1.85 ± 0.12a	2.75 ± 0.03b	2.20 ± 0.09b	1.83 ± 0.12a	1.70 ± 0.13a
Total coliforms	1.11 ± 0.21a	2.14 ± 0.05b	2.04 ± 0.14b	2.37 ± 0.03b	2.11 ± 0.21b
Faecal coliforms	˂ 1	˂ 1	˂ 1	˂ 1	˂ 1
Yeast and mould	1.85 ± 0.05a	2.97 ± 0.02c	2.11 ± 0.03b	1.87 ± 0.21a	2.17 ± 0.05b
*Staphylococcus aureus*	1.05 ± 0.22a	1.37 ± 0.34a	1.13 ± 0.01a	1.20 ± 0.03a	1.12 ± 0.09a

Mean ± Standard deviation; values with the same letter in the same line, are not significantly different at p < 0.05.

### Total phenolic content and the antioxidant activities of *P. butyracea* fruit pulp

Total phenolic content (TPC) of *P. butyracea* fruit pulp was expressed as gallic acid equivalent (GAE) per g of DM. The total phenolic content (TPC) and antioxidant activities (AA) of the pulps are shown in Table 6. The TPC values ranged between 21.54 and 25.98 mg/mL for all the fruit pulp samples. The TPC was higher (25.98 mg/mL) in fruit pulp collected in Kandi, while the lowest TPC (21.54 mg/mL) was recorded in fruit pulp sample from Toucountouna. The significant different (p ≤ 0.05) of TPC of the fruit pulp between parks may be explained by various maturity stages of the *P. butyracea* fruit. The variations of *P. butyracea* fruit TPC from various agroecologics zones could be attributed to climate and environnement factors namely rainfall and soil composition. The TPC of *P. butyracea* fruit pulp was higher than that found in honey by Ref.^30^, which varies from 0.23 to 0.73 mg GAE/g. While, The TPC of *P. butyracea* fruit pulp was lower compared to propolis values (22.80–77.50 mg GAE/g reported by Ref.^31^. In addition, the TPC of *P. butyracea* fruit pulp obtained in this study was higher than 226.25 mg/100 g reported by Ref.^24^ from fresh shea fruit pulp in Ghana.

**Table 6:** Parameters of free radical scavenging activity by DPPH and total phenolics of *P. butyracea* fruit pulp by park.

Parks	TPC (mg/mL dw)	EC_50_ (mg/mL DPPH)	% (DPPH) remaining
Bassila	23.22 ± 0.03b	0.012 ± 0.07a	15.07 ± 0.14c
Tchaourou	22.86 ± 0.01b	0.014 ± 0.21b	13.15 ± 0.09b
Kandi	25.98 ± 0.11c	0.010 ± 0.23d	10.83 ± 0.07a
Natitingou	23.52 ± 0.05b	0.011 ± 0.03c	12.74 ± 0.01b
Toucountouna	21.54 ± 0.03a	0.017 ± 0.11a	22.54 ± 1.17d
Ascorbic acid	–	0.0018 ± 0.0014	7.48 ± 0.01
Gallic acid	–	0.0020 ± 0.0001	9.83 ± 0.03

Mean ± Standard deviation; values with the same letter in the same column, are not significantly different at p < 0.05.

The antioxidant capacity of fruits and vegetables is an important indicator of health promoters, and many methods have been developed to evaluate this particular capacity^32^. In this study the anti-radical properties of the fruit pulps was performed by DPPH radical scavenging assay. EC_50_, the effective concentration of the extracts (mg antioxidant/mg DPPH) required to scavenge 50% of DPPH radical are presented. Significant difference in EC_50_ between the fruit pulp samples was observed (p ≤ 0.001). The pulp sample collected in Toucountouna park showed the highest EC_50_ value, whereas the pulp sample from Kandi was found to have the lowest EC_50_ value. A lower value would reflect greater antioxidant activity of sample. Moreover, the remaining DPPH in the fruit pulps ranged from 10.83 to 22.54%. This result suggests that *P. butyracea* fruit pulp plays an important role in the scavenging of free radical.

## Conclusion

This study showed that *P. butyracea* fruit pulp has high total phenolic content. In addition, *P. butyracea* fruit pulp is a valuable source of antioxidant pigments. The proximate and the mineral composition values obtained showed that *P. butyracea* fruit pulp is rich in total carbohydrates, crude fiber, calcium and iron. This fruit pulp is a good source of the minerals which are useful for the proper functioning of the body and important in the diet. Thus, consumption of *P. butyracea* fruit pulp is highly recommended in Benin.

## Data Availability

All the data supporting the results are included within the article.
